# Serum Bilirubin Levels and Extent of Symptomatic Intracranial Atherosclerotic Stenosis in Acute Ischemic Stroke: A Cross-Sectional Study

**DOI:** 10.3389/fneur.2021.714098

**Published:** 2021-08-26

**Authors:** Fang Yu, Lin Zhang, Di Liao, Yunfang Luo, Xianjing Feng, Zeyu Liu, Jian Xia

**Affiliations:** ^1^Department of Neurology, Xiangya Hospital, Central South University, Changsha, China; ^2^Clinical Research Center for Cerebrovascular Disease of Hunan Province, Central South University, Changsha, China; ^3^National Clinical Research Center for Geriatric Disorders, Xiangya Hospital, Central South University, Changsha, China

**Keywords:** ischemic stroke, symptomatic intracranial atherosclerotic stenosis, bilirubin, biomarkers, oxidative stress

## Abstract

**Background:** Bilirubin plays a paradoxical role in the pathological mechanism of stroke. To date, few clinical studies have investigated the effect of serum bilirubin on symptomatic intracranial atherosclerotic stenosis (sICAS). This study aims to evaluate the connection between serum bilirubin and sICAS.

**Methods:** From September 2015 to May 2020, 1,156 sICAS patients without hepatobiliary diseases admitted to our hospital were included. Patients were distributed into none-mild (0–49%), moderate (50–69%) and severe-occlusion sICAS groups (70–100%) by the degree of artery stenosis. Moderate and severe-occlusion sICAS patients were classified into three groups by the number of stenotic arteries (single-, two- and multiple-vessel stenosis). The relationship between serum bilirubin levels and sICAS was analyzed by logistic regression analysis.

**Results:** In univariable analyses, sICAS patients with severe and multiple atherosclerotic stenoses had lower levels of total bilirubin (Tbil), direct bilirubin (Dbil), and indirect bilirubin (Ibil). In multinomial logistic regression analyses, when compared with the highest tertile of bilirubin, lower levels of Tbil, Dbil, and Ibil showed higher risks of severe-occlusion sICAS (95% CI: 2.018–6.075 in tertile 1 for Tbil; 2.380–7.410 in tertile 1 for Dbil; 1.758–5.641 in tertile 1 for Ibil). Moreover, the logistic regression analyses showed that lower levels of Tbil, Dbil, and Ibil were related to multiple (≥3) atherosclerotic stenoses (95% CI: 2.365–5.298 in tertile 1 and 2.312–5.208 in tertile 2 for Tbil; 1.743–3.835 in tertile 1 and 1.416–3.144 in tertile 2 for Dbil; 2.361–5.345 in tertile 1 and 1.604–3.545 in tertile 2 for Ibil) when compared with tertile 3.

**Conclusions:** Our findings suggest that lower bilirubin levels may indicate severe and multiple intracranial atherosclerotic stenoses.

## Introduction

Symptomatic intracranial atherosclerosis (sICAS), a critical cause of ischemic stroke in China, refers to the stenosis ≥50% of one or more intracranial arteries (1). In Asia, ~50% of patients with transient ischemic attack (TIA) and 40% of patients with ischemic stroke (IS) have ICAS (2, 3). sICAS is a developing and dynamically changing disease with a high recurrence risk, causing a huge social burden. Traditional risk factors such as age, ethnicity, obesity, hypertension, diabetes, hyperlipidemia, smoking, and metabolic syndrome have been reported to be closely related to sICAS (4). However, the relationship between circulating biomarkers and sICAS is less explored.

Bilirubin is produced by heme catabolism, including total bilirubin (Tbli), indirect bilirubin (Ibil), and direct bilirubin (Dbil). In the past few decades, bilirubin has been considered as a potentially toxic metabolite, which could damage the central nervous system once passing through the blood-brain barrier (5, 6). However, subsequent evidence has shown that bilirubin plays a dual role in oxidative stress and it may be a protective factor for atherosclerosis (7, 8). Accumulating research suggests that bilirubin can inhibit the production of oxidized low-density lipoprotein (ox-LDL), increase the solubility of serum cholesterol, inhibit protein kinase C activity in human fibroblasts, and capture oxygen free radicals, thus inhibiting the progression of atherosclerosis (9–11).

Several clinical observations have indicated that high bilirubin concentrations could reduce the risk of stroke (12–14). Besides, a small number of studies have revealed the negative associations between high bilirubin levels and the occurrence of asymptomatic intracranial atherosclerosis (aICAS) (15) and extracranial atherosclerosis (16). However, research to date has not yet determined the relationship between bilirubin and sICAS.

Therefore, this research aimed to explore the relationship between serum bilirubin concentrations (including Tbil, Dbil, and Ibil) and sICAS in the Chinese Han Population.

## Methods

### Study Population

This study was a descriptive, retrospective, cross-sectional study. From September 2015 to May 2020, patients with TIA or acute ischemic stroke (AIS) caused by large artery atherosclerosis (LAA) within 14 days from symptom onset were enrolled from the Department of Neurology of Xiangya Hospital. The information of all patients was collected from the medical records. The diagnosis of AIS and TIA matched with the 2018 Chinese AIS guidelines (17). We assessed the stroke severity of AIS patients at admission using the National Institutes of Health Stroke Scale (NIHSS) score. All patients were subtyped by the Chinese ischemic stroke subclassification (CISS) system (18). We recruited 1,015 patients caused by intracranial atherosclerotic stenosis (stenosis ≥50%) and 141 patients attributed to atherosclerotic causes with none-mild intracranial stenosis (stenosis: 0–49%). Exclusion criteria were as follows: (1) under 18 years old; (2) other causes for TIA or IS such as small vessel occlusion or cardioembolism; (3) incomplete clinical information or laboratory tests; (4) patients with extracranial artery stenosis diagnosed by carotid contrast-enhanced magnetic resonance angiography (CE-MRA) or carotid computed tomography angiography (CTA) according to the methods used in the North American Symptomatic Carotid Endarterectomy Trial (19); (5) brain tumor, intracranial or systemic infection, congenital hypoplastic cerebrovascular disease, etc; (6) other diseases causing intracranial artery stenosis such as vascular malformation, moyamoya disease, artery dissection, vasculitis and syphilis (20); (7) suffering from hepatobiliary diseases (Tbil >34.2 μmol/L, alanine aminotransferase [ALT] ≥80 IU/L, aspartate aminotransferase ≥80 IU/L, serum albumin <3.5 g/dL) or other diseases that may affect bilirubin level such as Gilbert syndrome (Tbil >34.2 μmol/L, ALT <80 IU/L, aspartate aminotransferase <80 IU/L, γ-glutamyl transpeptidase <80 IU/L) (21). This study was approved by the Ethics Committees of Xiangya Hospital of Central South University, Changsha, Hunan Province, China (ethical approval number: 201503330). All patients or their family members signed the informed consent.

### Demographics and Risk Factors

The following clinical information was collected via questionnaires and physical examinations: age, sex, hypertension, diabetes mellitus, hyperlipemia, alcohol use, smoking duration, and the history of coronary artery disease. Hypertension was defined as systolic blood pressure ≥140 mmHg or diastolic blood pressure ≥90 mmHg or currently taking antihypertensive drugs (22). Diagnostic criteria for diabetes: random blood glucose ≥11.1 mmol/L, fasting blood glucose ≥7.0 mmol/L or using hypoglycemic drugs (23). Dyslipidemia was diagnosed as serum triglyceride ≥1.7 mmol/L, serum total cholesterol ≥5.2 mmol/L, serum low-density lipoprotein cholesterol ≥3.4 mmol/L, or serum high-density lipoprotein <1.0 mmol/L or using anti-hyperlipidemic drugs (24). Smoking was determined based on the self-report questionnaire at the time of admission, and the smoking amount was defined as pack-years in our study (25). The pack-years was measured based on the average smoking volume and the past and current smoking durations (25). All patients were divided into four groups according to smoking mount: group 1 (0, non-smoker), group 2 (0–15 pack-years), group 3 (15–30 pack-years), and group 4 (>30 pack-years) (25). The state of alcoholism was thought to be an average of more than 20 g of alcohol per day (26). Fasting overnight, the blood samples of all patients were collected the next morning after admission (within 14 days of stroke onset) and sent to the same laboratory department in our hospital. The data of white blood cell, Tbil, Dbil, Ibil, total cholesterol, triglyceride, high-density lipoprotein, low-density lipoprotein, fasting blood glucose, glycosylated hemoglobin A1c, uric acid, and homocysteine levels were derived from the medical records.

### Radiological Assessment

On admission, magnetic resonance imaging (MRI) and time of flight magnetic resonance angiography (TOF-MRA) were performed for most patients. Computed tomography (CT) and computed tomography angiography (CTA) were performed for patients with contraindications to MRI. In addition, CTA was performed when there were doubts about the results of MRA, and digital subtraction angiography (DSA) was performed when the results of MRA and CTA were inconsistent. Meanwhile, carotid CTA and carotid CE-MRA were used to exclude extracranial artery stenosis. All imaging data were evaluated by at least two neurologists with more than 5 years of experience. They knew nothing about the clinical information and reached a consensus. According to the results of MRA, CTA, DSA, or CE-MRA, ICAS was diagnosed as large intracranial artery stenosis (50–100%), including bilateral internal carotid artery (ICA), bilateral anterior cerebral artery (ACA), bilateral middle cerebral artery (MCA), bilateral posterior cerebral artery (PCA), bilateral basilar artery (BA) or bilateral vertebral artery (VA) (27). The degree of intracranial stenosis was assessed by MRA/CTA/DSA using Warfarin-Aspirin Symptomatic Intracranial Disease (WASID) method with reference to normal distal vessels (28). Patients with none-mild intracranial stenosis (stenosis: 0–49%) served as controls. In this study, participants were divided into none-mild group (0–49%), moderate group (50–69%), and severe-occlusion group (70–100%) based on the degree of artery stenosis. We then counted the number of intracranial stenotic arteries (stenosis ≥50%) of ICAS patients and classified patients into three groups (single-, two- and multiple-vessel stenosis) accordingly.

### Statistical Analysis

The statistical analysis was conducted using IBM SPSS Statistics 22.0 (Chicago, USA), and all data were expressed as frequency (%) and the median (interquartile range, IQR). Characteristics of the objects were compared with a chi-square (χ^2^) test for categorical variables and the Mann-Whitney *U* test or Kruskal-Wallis test for continuous variables. We also analyzed the association between bilirubin and sICAS in different groups according to the degree and number of vascular stenosis. Bilirubin levels were categorized into tertiles and the χ^2^ test for trends was used to analyze the dose-effect of Tbil, Dbil, and Ibil. Factors with *P* < 0.05 in univariate analysis and reported confounding risk factors were included in multivariate logistic regression analysis to evaluate the independent influence of bilirubin. Tbli, Dbil, and Ibil were tested separately to avoid interaction. We used multinomial logistic regression instead of ordinal polytomous logistic regression because the test of the parallel lines hypothesis was rejected. The results were shown by odds ratio (OR) and 95% confidence interval (CI). In addition, receiver operating characteristic curve (ROC) analysis was conducted by MedCalc software (MedCalc Inc., Mariakerke, Belgium) to determine the predictability of bilirubin for discriminating the extent of ICAS. *P* < 0.05 was considered significant.

## Results

### Clinical Characteristics of Patients With Ischemic Stroke

Clinical characteristics of all patients were presented in Table 1. A total of 1,156 subjects were finally included in the study. The average age of the participants was 61 (IQR, 53–68) years old and 65.5% were male. The median (IQR) of Tbil, Dbil, and Ibli levels was 10.34 (7.70–13.40), 4.50 (3.30–5.90), and 5.60 (4.91–7.18) μmol/L, separately.

**Table 1:** Baseline characteristics of patients with ischemic stroke.

**Characteristics**	**Value**
Age years [IQR]	61 [53–68]
Sex (male, *N*, %)	757 (65.5)
Hypertension (*N*, %)	872 (75.4)
Diabetes mellitus (*N*, %)	389 (33.7)
Hyperlipidemia (*N*, %)	521 (45.1)
CAD (*N*, %)	178 (15.4)
Smoking duration (pack-years) (*N*, %)	
Group 1 (0)	638 (55.2)
Group 2 (>0, ≤ 15)	107 (9.3)
Group 3 (>15, ≤ 30)	184 (15.9)
Group 4 (>30)	227 (19.6)
Drinking (*N*, %)	376 (32.5)
NIHSS [IQR]	4 [2–8]
SBP mmHg [IQR]	144 [132–158]
DBP mmHg [IQR]	84 [76–93]
WBC, ×10^9^/L [IQR]	6.80 [5.60–8.40]
Tbil, μmol/L [IQR]	10.34 [7.70–13.40]
Dbil, μmol/L [IQR]	4.50 [3.30–5.90]
Ibil, μmol/L [IQR]	5.60 [4.00–7.90]
FBG, mmol/L [IQR]	5.60 [4.91–7.18]
HbA1c, % [IQR]	5.80 [5.70–6.60]
TC, mmol/L [IQR]	4.37 [3.55–5.18]
TG, mmol/L [IQR]	1.55 [1.16–2.12]
HDL, mmol/L [IQR]	1.02 [0.87–1.22]
LDL, mmol/L [IQR]	2.66 [2.05–3.30]
UA, μmol/L [IQR]	316.20 [256.20–382.15]
HCY, μmol/L [IQR]	13.25 [11.11–16.35]

*IQR, Inter quartile range; CAD, coronary artery disease; NIHSS, National Institute of Health stroke scale; SBP, systolic blood pressure; DBP, diastolic blood pressure; WBC, white blood cell; Tbil, total bilirubin; Dbil, direct bilirubin; Ibil, indirect bilirubin; FBG, fasting blood glucose; HbA1c, glycosylated hemoglobin A1c; TC, total cholesterol; TG, triglyceride; HDL, high density lipoprotein; LDL, low density lipoprotein; UA, Uric acid; HCY, Homocysteine. Smoking duration: group 1 (0, non-smoker), group 2 (0–15 pack-years), group 3 (15–30 pack-years) and group 4 (>30 pack-years)*.

### Baseline Characteristics of the Study Population According to the Severity of ICAS

**Figure 1:**
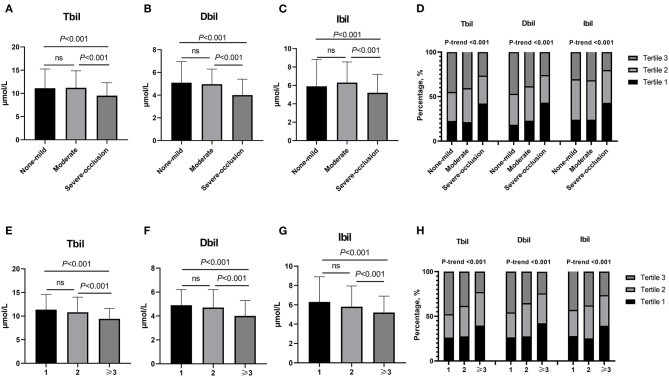
**(A–D)** Bilirubin levels in different groups stratified by the severity of ICAS (none-mild group: stenosis 0–49%, moderate group: stenosis 50–69% and severe-occlusion group: stenosis 70–100%). **(A)** Different Tbli levels among non-mild, moderate and severe-occlusion groups.Tertile 1: <8.6 μmol/L, Tertile 2: 8.6–12.1 μmol/L, Tertile 3: >12.1 μmol/L. **(B)** Different Dbli levels among non-mild, moderate and severe-occlusion groups. Tertile 1: <3.7 μmol/L, Tertile 2: 3.7–5.3 μmol/L, Tertile 3: >5.3 μmol/L. **(C)** Different Ibli levels among non-mild, moderate and severe-occlusion groups. Tertile 1: <4.6 μmol/L, Tertile 2: 4.6–7.9 μmol/L, Tertile 3: >7.9 μmol/L. **(D)** The different percentages of Tbli, Dbli and Ibli tertiles among non-mild, moderate and severe-occlusion groups. **(E**–**H)** Different bilirubin levels in groups according to the number of stenoses (≥50%) (*n* = 1, 2, and ≥3). **(E)** Different Tbli levels among groups. Tertile 1: <8.4 μmol/L, Tertile 2: 8.4–11.9 μmol/L, Tertile 3: >11.9 μmol/L. **(F)** Different Dbli levels among groups. Tertile 1: <3.6 μmol/L, Tertile 2: 3.6–5.1 μmol/L, Tertile 3: >5.1 μmol/L. **(G)** Different Ibli levels among groups. Tertile 1: <4.5 μmol/L, Tertile 2: 4.5–6.9 μmol/L, Tertile 3: >6.9 μmol/L. **(H)** The different percentages of Tbli, Dbli and Ibli tertiles in groups according to the number of stenoses (≥50%) (*n* = 1, 2, and ≥3). *n* = 1, single-vessel stenosis; *n* = 2, two-vessel stenosis; *n* ≥ 3, multiple-vessel stenosis. ICAS, intracranial atherosclerotic stenosis; Tbil, total bilirubin; Dbil, direct bilirubin; Ibil, indirect bilirubin.

According to the degree of intracranial artery stenosis, participants were divided into none-mild group (0–49%, *n* = 141), moderate group (50–69%, *n* = 357), and severe-occlusion group (70–100%, *n* = 658). Baseline clinical characteristics and laboratory tests of these 1,156 objects were shown in Table 2. There were significant differences among these three groups in sex, hypertension, hyperlipidemia, systolic blood pressure, diastolic blood pressure, and the levels of Tbil, Dbil, Ibil, uric acid, and homocysteine. Moreover, Figures 1A–C illustrates the median concentrations of Tbil, Dbil and Ibil in different groups according to the severity of ICAS. Lower bilirubin levels were found in the severe-occlusion group compared with the none-mild or moderate group. Bilirubin levels were categorized into tertiles, and the linear trends across the three categories were tested by chi-square linear trend test. The chi-square linear trend test yielded a significant result (*P*-trends < 0.001) (Figure 1D), mostly due to the significant difference in bilirubin levels between the moderate and severe-occlusion ICAS groups. The serum bilirubin levels in patients with none-mild ICAS were similar compared with patients presenting with moderate ICAS. We tried to find clear cutoff values by ROC analyses to discriminate the severity of ICAS, however, the performance of these models was rather poor with AUCs of 0.627–0.640. The results were shown in Supplementary Figure 1A and Supplementary Table 1.

**Table 2:** Baseline characteristics of the study population according to the severity of ICAS.

**Characteristics**	**None-mild (*n* = 141)**	**Moderate (*n* = 357)**	**Severe-occlusion (*n* = 658)**	***P-*value**
Age, years [IQR]	62 [53–70]	62 [54–69]	61 [52–68]	0.054
Sex (male, *N*, %)	103 (73.0)	241 (67.5)	413 (62.8)	0.041
Hypertension (*N*, %)	99 (70.2%)	287 (80.4)	486 (73.9)	0.021
Diabetes mellitus (*N*, %)	43 (30.5)	131 (36.7)	215 (32.7)	0.303
HbA1c (yes), % [IQR]	7.20 [6.30–9.20]	7.10 [6.10–8.50]	7.30 [6.30–8.60]	0.557
HbA1c (no), % [IQR]	5.80 [5.60–6.03]	5.80 [5.50–5.80]	5.80 [5.50–5.80]	0.167
Hyperlipidemia (*N*, %)	37 (26.2%)	163 (45.7)	321 (48.8)	<0.001
LDL (yes), mmol/L [IQR]	3.01 [2.02–4.14]	3.13 [2.32–3.69]	2.95 [2.24–3.69]	0.794
LDL (no), mmol/L [IQR]	2.40 [2.00–3.13]	2.58 [2.02–3.04]	2.44 [1.99–2.90]	0.217
CAD (*N*, %)	14 (9.9)	58 (16.2)	105 (16.1)	0.158
Smoking duration (pack-years) (*N*, %)				0.054
Group 1 (0)	70 (49.6)	187 (52.4)	381 (57.9)	
Group 2 (>0, ≤ 15)	10 (7.1)	42 (11.8)	55 (8.4)	
Group 3 (>15, ≤ 30)	33 (23.4)	52 (14.6)	99 (15.0)	
Group 4 (>30)	28 (19.9)	76 (21.3)	123 (18.7)	
Drinking (*N*, %)	48 (34.0)	116 (32.5)	212 (32.2)	0.916
NIHSS [IQR]	4 [2–7]	4 [2–7]	4 [2–8]	0.143
SBP mmHg [IQR]	143 [134–158]	148 [134–162]	143 [131–155]	0.002
DBP mmHg [IQR]	87 [79–92]	85 [77–94]	82 [75–92]	0.008
WBC, ×10^9^/L [IQR]	6.55 [5.60–8.00]	6.80 [5.60–8.50]	6.85 [5.60–8.40]	0.599
Tbil, μmol/L [IQR]	11.10 [8.85–15.25]	11.20 [9.10–14.85]	9.50 [6.90–12.30]	<0.001
Dbil, μmol/L [IQR]	5.10 [4.05–6.95]	4.96 [3.80–6.30]	4.00 [3.00–5.40]	<0.001
Ibil, μmol/L [IQR]	5.90 [4.70–8.80]	6.30 [4.70–8.55]	5.20 [3.60–7.20]	<0.001
FBG, mmol/L [IQR]	5.50 [4.54–7.56]	5.72 [5.07–7.24]	5.56 [4.90–7.08]	0.053
HbA1c, % [IQR]	5.90 [5.80–6.80]	5.80 [5.70–6.55]	5.80 [5.60–6.50]	0.210
TC, mmol/L [IQR]	4.44 [3.51–5.42]	4.44 [3.62–5.15]	4.30 [3.50–5.17]	0.515
TG, mmol/L [IQR]	1.59 [1.16–2.18]	1.50 [1.15–2.05]	1.57 [1.18–2.22]	0.446
HDL, mmol/L [IQR]	1.04 [0.87–2.20]	1.02 [0.88–1.21]	1.01 [0.87–1.23]	0.851
LDL, mmol/L [IQR]	2.51 [2.00–3.33]	2.78 [2.10–3.32]	2.61 [2.08–3.29]	0.176
UA, μmol/L [IQR]	333.10 [269.30–403.03]	333.95 [273.03–390.08]	306.90 [250.40–371.80]	<0.001
HCY, μmol/L [IQR]	13.50 [11.72–16.06]	13.57 [11.28–16.46]	12.76 [10.82–16.20]	0.031

*None-mild: stenosis 0–49%, moderate: stenosis 50–69%, severe-occlusion group: stenosis 70–100%. ICAS, intracranial atherosclerotic stenosis; IQR, Inter quartile range; HbA1c, glycosylated hemoglobin A1c; LDL, low density lipoprotein; CAD, coronary artery disease; NIHSS, National Institute of Health stroke scale; SBP, systolic blood pressure; DBP, diastolic blood pressure; WBC, white blood cell; Tbil, total bilirubin; Dbil, direct bilirubin; Ibil, indirect bilirubin; FBG, fasting blood glucose; TC, total cholesterol; TG, triglyceride; HDL, high density lipoprotein; LDL, low density lipoprotein; UA, uric acid; HCY, homocysteine. Smoking duration: group 1 (0, non-smoker), group 2 (0–15 pack-years), group 3 (15–30 pack-years) and group 4 (>30 pack-years)*.

### Baseline Characteristics of the Study Population Based on the Number of Stenotic Arteries

Based on the number of stenotic arteries, patients were stratified into three groups: single-vessel stenosis (*n* = 294), two-vessel stenosis (*n* = 193) and multiple-vessel stenosis (*n* = 528). Table 3 provides the specific clinical information and laboratory results. There were significant differences among the three groups in age, hypertension, diabetes, hyperlipidemia, Tbil, Dbil, Ibil, fasting blood glucose, and glycosylated hemoglobin A1c. In addition, lower bilirubin levels were found in the multiple-vessel stenosis group compared with the single- and two-vessel stenosis groups (as shown in Figures 1E–G). The chi-square linear trend test yielded a significant result (*P*-trends < 0.001) (Figure 1H), mostly due to the significant difference in bilirubin levels between the two- and multiple-vessel stenosis groups. ROC analyses were used to differentiate the number of stenotic arteries in patients with ICAS, unfortunately, the ROC methods demonstrated poor discriminatory capability for constructing cutoff values (AUCs: 0.600, 0.607, and 0.615). The results were presented in the Supplementary Figure 1B and Supplementary Table 1.

**Table 3:** Baseline characteristics of the participants according to the number of stenotic arteries.

**Characteristics**	**1 (*n* = 294)**	**2 (*n* = 193)**	**≥3 (*n* = 528)**	***P*-value**
Age years [IQR]	57 [48–66]	62 [53–69]	63 [54–69]	<0.001
Sex (male, *N*, %)	204 (69.4)	127 (65.8)	323 (661.2)	0.056
Hypertension (*N*, %)	191 (65.0)	154 (79.8)	428 (81.1)	<0.001
Diabetes mellitus (*N*, %)	78 (26.5)	69 (35.8)	199 (37.7)	0.005
HbA1c (yes), % [IQR]	6.65 [5.80–7.80]	7.20 [6.20–8.70]	7.40 [6.40–8.60]	0.002
HbA1c (no), % [IQR]	5.80 [5.50–5.80]	5.80 [5.60–5.90]	5.80 [5.50–5.90]	0.183
Hyperlipidemia (*N*, %)	160 (54.4)	77 (39.9)	247 (46.8)	0.006
LDL (yes), mmol/L [IQR]	3.06 [2.25–3.70]	3.06 [2.20–3.55]	2.95 [2.28–3.72]	0.896
LDL (no), mmol/L [IQR]	2.45 [1.94–2.94]	2.56 [2.11–2.94]	2.47 [2.02–2.97]	0.400
CAD (*N*, %)	42 (14.3)	34 (17.6)	88 (16.7)	0.559
Smoking duration (pack-years) (*N*, %)				0.092
Group 1 (0)	152 (51.7)	105 (54.4)	311 (58.9)	
Group 2 (>0, ≤ 15)	31 (10.5)	17 (8.8)	49 (9.3)	
Group 3 (>15, ≤ 30)	40 (13.6)	27 (14.0)	84 (15.9)	
Group 4 (>30)	71 (24.1)	44 (22.8)	84 (15.9)	
Drinking (*N*, %)	94 (32.0)	71 (36.8)	163 (30.9)	0.319
NIHSS [IQR]	5 [2–8]	5 [2–8]	4 [2–7]	0.151
SBP mmHg [IQR]	144 [128–155]	143 [134–158]	145 [133–159]	0.078
DBP mmHg [IQR]	84 [76–95]	85 [77–93]	82 [75–91]	0.128
WBC, ×10^9^ /L [IQR]	6.90 [5.60–8.50]	7.20 [5.60–8.78]	6.70 [5.60–8.20]	0.220
Tbil, μmol/L [IQR]	11.35 [8.10–14.60]	10.80 [8.30–14.00]	9.40 [7.03–11.60]	<0.001
Dbil, μmol/L [IQR]	4.90 [3.58–6.20]	4.70 [3.60–6.20]	4.00 [3.00–5.30]	<0.001
Ibil, μmol/L [IQR]	6.30 [4.40–8.90]	5.80 [4.45–7.95]	5.20 [3.70–6.90]	<0.001
FBG, mmol/L [IQR]	5.50 [4.91–6.69]	5.92 [5.09–7.53]	5.67 [4.93–7.22]	0.032
HbA1c, % [IQR]	5.80 [5.60–6.10]	5.80 [5.70–6.95]	5.80 [5.70–6.78]	<0.001
TC, mmol/L [IQR]	4.38 [3.50–5.30]	4.41 [3.65–5.14]	4.34 [3.57–5.11]	0.498
TG, mmol/L [IQR]	1.63 [1.21–2.20]	1.50 [1.12–2.06]	1.54 [1.16–2.11]	0.218
HDL, mmol/L [IQR]	1.02 [0.88–1.24]	1.02 [0.87–1.25]	1.01 [0.87–1.19]	0.481
LDL, mmol/L [IQR]	2.71 [2.07–3.41]	2.75 [2.17–3.27]	2.60 [2.08–3.29]	0.760
UA, μmol/L [IQR]	316.55 [252.60–375.80]	315.45 [259.48–387.60]	310.30 [256.05–375.55]	0.858
HCY, μmol/L [IQR]	13.56 [10.96–16.54]	12.52 [11.13–14.90]	13.01 [10.93–16.69]	0.350

*1, single-vessel stenosis; 2, two-vessel stenosis; ≥3, multiple-vessel stenosis. IQR, Inter quartile range; HbA1c, glycosylated hemoglobin A1c; CAD, coronary artery disease; NIHSS, National Institute of Health stroke scale; SBP, systolic blood pressure; DBP, diastolic blood pressure; WBC, white blood cell; Tbil, total bilirubin; Dbil, direct bilirubin; Ibil, indirect bilirubin; FBG, fasting blood glucose; TC, total cholesterol; TG, triglyceride; HDL, high density lipoprotein; LDL, low density lipoprotein; UA, uric acid; HCY, homocysteine. Smoking duration: group 1 (0, non-smoker), group 2 (0–15 pack-years), group 3 (15–30 pack-years) and group 4 (>30 pack-years)*.

### Multivariate Logistic Regression Analyses for Predictors of Severe ICAS

The findings obtained from the multivariate logistic regression analysis are presented in Table 4. The variables with *P* < 0.05 in univariate analysis in Table 2 and other reported confounding factors were input into the multivariate model. Tbil, Dbil, and Ibil did not have significant influence on the degree of ICAS when comparing the group with moderate ICAS with the none-mild ICAS group. When comparing the severe-occlusion and none-mild groups, lower Tbil, Dbil, and Ibil levels were found to be independent factors (OR: 3.502, 95% CI: 2.018–6.075, *P* < 0.001 in tertile 1 for Tbil; OR: 4.199, 95% CI: 2.380–7.410, *P* < 0.001 in tertile 1 for Dbil; OR: 3.149, 95% CI: 1.758–5.641, *P* < 0.001 in tertile 1 for Ibil) compared to the highest levels of bilirubin.

**Table 4:** Multivariate logistic regression analyses for predictors of severe-occlusion of ICAS.

	**Moderate vs. None-mild**		**Severe-occlusion vs. None-mild**		**Moderate vs. None-mild**		**Severe-occlusion vs. None-mild**		**Moderate vs. None-mild**		**Severe-occlusion vs. None-mild**	
	***P*-value**	**OR (95% CI)**	***P*-value**	**OR (95% CI)**	***P*-value**	**OR (95% CI)**	***P*-value**	**OR (95% CI)**	***P*-value**	**OR (95% CI)**	***P*-value**	**OR (95% CI)**
Age, years	0.768	0.997 (0.975–1.019)	0.116	0.984 (0.964–1.004)	0.803	0.997 (0.976–1.019)	0.118	0.984 (0.964–1.004)	0.775	0.997 (0.975–1.019)	0.129	0.984 (0.964–1.005)
Sex, (Female vs. male)	0.416	0.771 (0.412–1.444)	0.926	1.029 (0.569–1.860)	0.538	0.820 (0.437–1.541)	0.908	1.036 (0.571–1.878)	0.433	0.778 (0.415–1.457)	0.967	1.013 (0.561–1.829)
Hypertension	0.045	1.773 (1.013–3.104)	0.236	1.364 (0.816–2.280)	0.062	1.700 (0.973–2.972)	0.238	1.362 (0.815–2.275)	0.042	1.788 (1.022–3.127)	0.206	1.392 (0.834–2.322)
Diabetes mellitus	0.376	1.245 (0.767–2.020)	0.999	1.000 (0.629–1.590)	0.450	1.206 (0.743–1.957)	0.875	0.963 (0.605–1.534)	0.356	1.255 (0.774–2.034)	0.884	1.035 (0.652–1.642)
Hyperlipidemia	0.001	2.270 (1.411–3.653)	<0.001	2.792 (1.772–4.399)	0.001	2.257 (1.403–3.631)	<0.001	2.548 (1.616–4.015)	0.001	2.272 (1.410–3.659)	<0.001	2.806 (1.781–4.422)
**Smoking duration**												
Group 1 (0)	Reference		Reference		Reference		Reference			Reference		
Group 2 (>0, ≤ 15)	0.087	2.158 (0.895–5.203)	0.991	1.005 (0.422–2.393)	0.098	2.106 (0.872–5.084)	0.860	1.081 (0.454–2.576)	0.092	2.131 (0.884–5.140)	0.979	1.012 (0.426–2.404)
Group 3 (>15, ≤ 30)	0.324	0.715 (0.367–1.393)	0.101	0.596 (0.321–1.106)	0.326	0.716 (0.367–1.396)	0.112	0.604 (0.325–1.125)	0.340	0.723 (0.371–1.408)	0.128	0.619 (0.334–1.147)
Group 4 (>30)	0.405	1.333 (0.678–2.617)	0.816	0.926 (0.486–1.764)	0.408	1.331 (0.676–2.623)	0.994	0.997 (0.523–1.903)	0.421	1.319 (0.672–2.591)	0.754	0.902 (0.475–1.716)
SBP, mmHg	0.228	1.009 (0.995–1.023)	0.767	1.002 (0.989–1.016)	0.224	1.009 (0.995–1.023)	0.925	1.001 (0.987–1.014)	0.204	1.009 (0.995–1.024)	0.733	1.002 (0.989–1.016)
DBP, mmHg	0.039	0.976 (0.954–0.999)	0.014	0.973 (0.952–0.994)	0.044	0.977 (0.955–0.999)	0.017	0.974 (0.953–0.995)	0.031	0.975 (0.953–0.998)	0.013	0.973 (0.952–0.994)
UA, μmol/L	0.931	1.000 (0.998–1.002)	0.015	0.997 (0.995–0.999)	0.785	1.000 (0.997–1.002)	0.010	0.997 (0.995–0.999)	0.931	1.000 (0.998–1.002)	0.020	0.997 (0.995–1.000)
HCY, μmol/L	0.696	0.996 (0.979–1.014)	0.961	1.000 (0.985–1.016)	0.775	0.997 (0.980–1.015)	0.905	1.001 (0.986–1.017)	0.675	0.996 (0.980–1.013)	0.974	1.000 (0.985–1.015)
Tbil, μmol/L	P-trend: 0.678		P-trend: <0.001									
Tertile1 (<8.6)	0.737	1.108 (0.611–2.009)	<0.001	3.502 (2.018–6.075)								
Tertile2 (8.6–12.1)	0.518	1.182 (0.712–1.961)	0.226	1.354 (0.829–2.213)								
Tertile3 (>12.1)	Reference		Reference									
Dbil, μmol/L					P-trend: 0.145		P-trend: <0.001					
Tertile 1 (<3.7)					0.161	1.544 (0.841–2.835)	<0.001	4.199 (2.380–7.410)				
Tertile 2 (3.7–5.3)					0.198	1.393 (0.841–2.307)	0.061	1.592 (0.979–2.589)				
Tertile 3 (>5.3)					Reference		Reference					
Ibil, μmol/L									P-trend: 0.751		P-trend: <0.001	
Tertile 1 (<4.6)									0.736	1.113 (0.598–2.069)	<0.001	3.149 (1.758–5.641)
Tertile 2 (4.6–7.9)									0.937	1.021 (0.605–1.724)	0.354	1.271 (0.765–2.113)
Tertile 3 (>7.9)									Reference		Reference	

*None-mild: stenosis 0–49%, moderate: stenosis 50–69%, severe-occlusion group: stenosis 70–100%. ICAS, intracranial atherosclerotic stenosis; SBP, systolic blood pressure; DBP, diastolic blood pressure; UA, uric acid; HCY, homocysteine; Tbil, total bilirubin; Dbil, direct bilirubin; Ibil, indirect bilirubin. Smoking duration: group 1 (0, non-smoker), group 2 (0–15 pack-years), group 3 (15–30 pack-years) and group 4 (>30 pack-years)*.

### Multivariate Logistic Regression Analyses for Predictors of Multi-Stenosis of ICAS

Table 5 shows the results of multinomial logistic regression analysis. We compared two- and multiple-vessel stenosis groups separately, with single-vessel stenosis group as the control. Using tertile 3 as a reference, we found that lower levels of Tbil, Dbil, and Ibil were related to multiple (two-vessel stenosis or multiple-vessel stenosis) atherosclerotic stenosis (when two- vs. single-vessel stenosis, OR: 2.052, 95% CI: 1.270–3.314, *P* = 0.003 in tertile 2 for Tbil; OR: 1.866, 95% CI: 1.167–2.986, *P* = 0.009 in tertile 2 for Ibil. When multiple- vs. single-vessel stenosis, OR: 3.540, 95% CI: 2.365–5.298, *P* <0.001 in tertile 1 and OR: 3.470, 95% CI: 2.312–5.208, *P* < 0.001 in tertile 2 for Tbil; OR: 2.585, 95% CI: 1.734–3.835, *P* < 0.001 in tertile 1 and OR: 2.110, 95% CI: 1.416–3.144, *P* < 0.001 in tertile 2 for Dbil; OR: 3.552, 95% CI: 2.361–5.345, *P* < 0.001 in tertile 1 and OR: 2.384, 95% CI: 1.604–3.545, *P* < 0.001 in tertile 2 for Ibil).

**Table 5:** Multivariate logistic regression analyses for predictors of multi-stenosis of ICAS.

	**2 vs. 1**		**≥3 vs. 1**		**2 vs. 1**		**≥3 vs. 1**		**2 vs. 1**		**≥3 vs. 1**	
	***P*-value**	**OR (95% CI)**	***P*-value**	**OR (95% CI)**	***P*-value**	**OR (95% CI)**	***P*-value**	**OR (95% CI)**	***P*-value**	**OR (95% CI)**	***P*-value**	**OR (95% CI)**
Age, years	0.012	1.025 (1.006–1.045)	<0.001	1.046 (1.029–1.062)	0.017	1.024 (1.004–1.043)	<0.001	1.043 (1.027–1.059)	0.013	1.024 (1.005–1.044)	<0.001	1.044 (1.028–1.061)
Sex, (Female vs. male)	0.943	0.980 (0.565–1.700)	0.663	0.905 (0.577–1.419)	0.875	0.957 (0.553–1.656)	0.564	0.878 (0.564–1.366)	0.905	0.967 (0.558–1.676)	0.624	0.894 (0.573–1.397)
Hypertension	0.010	1.863 (1.159–2.997)	0.001	1.931 (1.319–2.827)	0.009	1.884 (1.171–3.029)	<0.001	1.962 (1.348–2.855)	0.013	1.826 (1.136–2.934)	0.001	1.930 (1.321–2.818)
Diabetes mellitus	0.729	1.101 (0.640–1.895)	0.561	1.143 (0.729–1.793)	0.847	1.055 (0.614–1.812)	0.693	1.094 (0.701–1.706)	0.795	1.075 (0.624–1.850)	0.626	1.118 (0.714–1.750)
Hyperlipidemia	0.003	0.525 (0.344–0.801)	0.135	0.769 (0.545–1.085)	0.003	0.531 (0.349–0.807)	0.118	0.764 (0.544–1.071)	0.003	0.526 (0.345–0.802)	0.160	0.782 (0.556–1.101)
**Smoking duration**												
Group 1 (0)	Reference		Reference		Reference		Reference		Reference		Reference	
Group 2 (>0, ≤ 15)	0.913	0.956 (0.427–2.142)	0.833	1.071 (0.564–2.036)	0.851	0.925 (0.414–2.070)	0.830	1.072 (0.569–2.019)	0.962	0.981 (0.438–2.194)	0.819	1.077 (0.569–2.038)
Group 3 (>15, ≤ 30)	0.739	1.124 (0.566–2.231)	0.421	1.259 (0.719–2.204)	0.693	1.147 (0.579–2.272)	0.357	1.296 (0.746–2.253)	0.669	1.161 (0.586–2.299)	0.324	1.322 (0.759–2.302)
Group 4 (>30)	0.925	1.029 (0.569–1861)	0.104	0.660 (0.399–1.089)	0.977	1.009 (0.560–1.818)	0.099	0.661 (0.404–1.081)	0.886	1.044 (0.578–1.888)	0.087	0.648 (0.394–1.066)
FBG, mmol/L	0.580	1.027 (0.934–1.131)	0.440	0.967 (0.887–1.054)	0.557	1.029 (0.936–1.131)	0.454	0.968 (0.890–1.054)	0.516	1.033 (0.937–1.137)	0.470	0.969 (0.890–1.055)
HbA1c, %	0.121	1.174 (0.959–1.436)	0.014	1.244 (1.045–1.481)	0.105	1.181 (0.966–1.444)	0.012	1.247 (1.050–1.482)	0.124	1.173 (0.957–1.437)	0.015	1.240 (1.042–1.475)
LDL, mmol/L	0.795	0.969 (0.765–1.228)	0.668	0.959 (0.792–1.161)	0.592	0.938 (0.741–1.186)	0.182	0.880 (0.729–1.062)	0.822	0.973 (0.766–1.236)	0.998	1.000 (0.825–1.213)
Tbil, μmol/L	P-trend: 0.101		P-trend: <0.001									
Tertile 1 (<8.4)	0.120	1.484 (0.902–2.441)	<0.001	3.540 (2.365–5.298)								
Tertile 2 (8.4-11.9)	0.003	2.052 (1.270–3.314)	<0.001	3.470 (2.312–5.208)								
Tertile 3 (>11.9)	Reference		Reference									
Dbil, μmol/L					P-trend: 0.610		P-trend: <0.001					
Tertile 1 (<3.6)					0.617	1.136 (0.689–1.874)	<0.001	2.585 (1.743–3.835)				
Tertile 2 (3.6–5.1)					0.068	1.552 (0.967–2.491)	<0.001	2.110 (1.416–3.144)				
Tertile 3 (>5.1)					Reference		Reference					
Ibil, μmol/L									P-trend: 0.136		P-trend: <0.001	
Tertile 1 (<4.5)									0.145	1.461 (0.877–2.434)	<0.001	3.552 (2.361–5.345)
Tertile 2 (4.5–6.9)									0.009	1.866 (1.167–2.986)	<0.001	2.384 (1.604–3.545)
Tertile 3 (>6.9)									Reference		Reference	

*1, single-vessel stenosis; 2, two-vessel stenosis; ≥3, multiple-vessel stenosis. ICAS, intracranial atherosclerotic stenosis; FBG, fasting blood glucose; HbA1c, glycosylated hemoglobin A1c; LDL, low density lipoprotein; Tbil, total bilirubin; Dbil, direct bilirubin; Ibil, indirect bilirubin. Smoking duration: group 1 (0, non-smoker), group 2 (0–15 pack-years), group 3 (15–30 pack-years) and group 4 (>30 pack-years)*.

## Discussion

The present study indicates that patients with severe and multiple sICAS had significantly lower serum bilirubin levels, even after adjusting for confounding factors such as age, sex, smoking status, hypertension, diabetes and hyperlipidemia.

ICAS is a leading cause of stroke occurrence and recurrence worldwide and is associated with higher risk for ischemic stroke and death. Since atherosclerosis is a chronic disease mediated by endothelial dysfunction, lipid deposition and inflammation, oxidative stress might play a crucial role in the pathological processes of sICAS (29–32). Prior studies have shown that endothelial dysfunction acts in the preclinical development of atherosclerosis, and inflammation could increase the vulnerability of plaques. Under oxidative stress, LDL could be transformed into oxidized low-density lipoprotein (ox-LDL), and vascular endothelial dysfunction and increased permeability could promote the deposition of ox-LDL in the intima (33). Furthermore, the accumulated ox-LDL contributes to the initiation of inflammatory reactions, infiltration of monocytes and T cells, and accumulation of extracellular matrix (34). T cells could recognize antigens and initiate the Type-1 immunity, causing local inflammation and plaque growth, leading to gradual narrowing of blood vessels and ICAS development (34).

Bilirubin has antioxidant and anti-inflammatory activities, reported to be inversely correlated with asymptomatic intracranial atherosclerosis (15, 35, 36). The mechanisms by which bilirubin functions in ICAS remains unclear, but prior studies indicate that bilirubin could inhibit atherosclerosis in several ways. First, reactive oxygen species promote lipid peroxidation, endothelial cell injury, smooth muscle cell proliferation and migration, inflammatory factor expression, and foam cell formation, leading to atherosclerosis and ultimately cerebral ischemia (37–39). Bilirubin, as the main end-product of heme metabolism, can scavenge free radicals and reduce the production of reactive oxygen species, thereby reducing the progression of atherosclerosis (40, 41). Second, a study by Vachharajani et al. showed that bilirubin could down-regulate the expression of P- and E- selectin induced by endotoxin, thus accounting for its anti-platelet aggregation effect (42). It has also been demonstrated that high concentrations of Ibil, as seen in Gilbert syndrome, could inhibit platelet aggregation induced by collagen and adenosine diphosphate (43). Third, bilirubin is negatively correlated with inflammatory markers, such as C-reactive protein, neutrophil-leukocyte ratio, and red cell distribution width, indicating that bilirubin could reduce pro-inflammatory cytokines and might also inhibit the inherent inflammatory process of atherosclerosis (44). Fourth, bilirubin could dissolve and transport cholesterol. Patients with hereditary diseases associated with elevated bilirubin levels have increased high-density lipoprotein/LDL ratio and decreased apolipoprotein B/apolipoprotein A-1 and total cholesterol levels (43). Fifth, previous research has established that bilirubin could delay the progression of atherosclerosis and improve vessel wall elasticity by down-regulating matrix metalloproteinase (45–47).

Accumulating clinical evidence proves that bilirubin has a protective impact on the carotid artery (48), cardiovascular system (9), and peripheral blood vessels (49). Nevertheless, there has been little discussion about the association between bilirubin and ICAS. In 2020, a population-based cross-sectional study pointed that serum Tbil, Dbil, and Ibil levels were negatively interrelated with aICAS, which is in accordance with our results (15). However, this paper focused on the connection between bilirubin (including Tbil, Dbil, and Ibil) and aICAS rather than sICAS. In addition, some scholars have examined the influence of serum Tbil on cerebral atherosclerosis and cerebral small vessel disease in the same subject and found that serum Tbil levels were negatively correlated with cerebral atherosclerosis (16). A study by Chen et al. revealed that Ibil concentrations increase with the exacerbation of intracranial or extracranial atherosclerotic stenosis, but decrease in patients with cranial vascular occlusion. In addition, there was no correlation between serum Tbil and Dbil levels and ICAS in their study (50). This is inconsistent with our results. A possible explanation for this might be that only 189 patients were recruited and they were divided into normal, mild (<50%), moderate (50–69%), severe (70–99%) and occlusion groups in their research. Meanwhile, the patients' gender, hypertension, smoking and alcohol consumption histories in each group were not completely matched in their study.

Hyperlipidemia is a well-established risk factor of macroangiopathy. However, our results showed that the presence of hyperlipidemia is a negative predictor for multi-stenosis of ICAS in the regression model of Table 5. One possible explanation for this paradox could be that the variable “hyperlipidemia” (yes/no) is rather imprecise when accounting a patient's risk for atherosclerosis. In our study, hyperlipidemia cases also included patients who had a history of hyperlipidemia or were currently receiving anti-hyperlipidemia therapy, while their present blood lipid levels may be within normal ranges. This was also supported by the unremarkable differences in LDL concentrations among the three groups (single-, two- and multiple-vessel stenosis) in patients with diagnosis of hyperlipidemia.

In this study, the bilirubin levels were significantly lower in patients with severe-occlusion or multiple-vessel stenosis, however, no clear linear dose-effect relationship between bilirubin levels and the extent of ICAS could be extrapolated from our data. The potential reasons are that the sample size of the none-mild group is relatively small and MRA was used to assess the degree of ICAS in the majority of patients, which is not the gold standard. MRA may amplify the extent of ICAS due to vascular tortuosity and various artifacts, thus causing diagnostic bias. Further research on the dose-effect-relationship between bilirubin levels and ICAS is needed.

There were some limitations in this study. First, it is a hospital-based, descriptive, retrospective cross-sectional study, and the results are unable to demonstrate a causal relationship between sICAS and serum bilirubin levels. Second, the participants were recruited from a single-center, so one should be cautious in inferring the results to other populations. Third, we only recorded the baseline levels of serum bilirubin, which might have a dynamic change during the development of ischemic stroke. Fourth, the evaluation of sICAS was based on different imaging methods, which might lead to diagnostic bias. Further studies, preferably with multicenter design, are needed to be conducted to confirm our findings.

## Conclusion

In conclusion, we found that lower bilirubin levels might indicate severe and multiple atherosclerotic stenoses of patients with sICAS in a Chinese Han population.

## Data Availability Statement

The raw data supporting the conclusions of this article will be made available by the authors, without undue reservation.

## Ethics Statement

The studies involving human participants were reviewed and approved by Xiangya Hospital, Central South University. The patients/participants provided their written informed consent to participate in this study.

## Author Contributions

FY and LZ: methodology and writing—original draft preparation. ZL, YL, DL, and XF: investigation and data curation. JX: conceptualization and writing—reviewing and editing. All authors contributed to the article and approved the submitted version.

## Conflict of Interest

The authors declare that the research was conducted in the absence of any commercial or financial relationships that could be construed as a potential conflict of interest.

## Publisher's Note

All claims expressed in this article are solely those of the authors and do not necessarily represent those of their affiliated organizations, or those of the publisher, the editors and the reviewers. Any product that may be evaluated in this article, or claim that may be made by its manufacturer, is not guaranteed or endorsed by the publisher.
