# Supplementary material for: Serum Bilirubin Levels and Extent of Symptomatic Intracranial Atherosclerotic Stenosis in Acute Ischemic Stroke: A Cross-Sectional Study
Source: Front Neurol. 2021 Aug 26;12:714098. doi: 10.3389/fneur.2021.714098 (PMC8427197; doi:10.3389/fneur.2021.714098)
Supplement: Supplementary file 1 [file Data_Sheet_1.PDF]

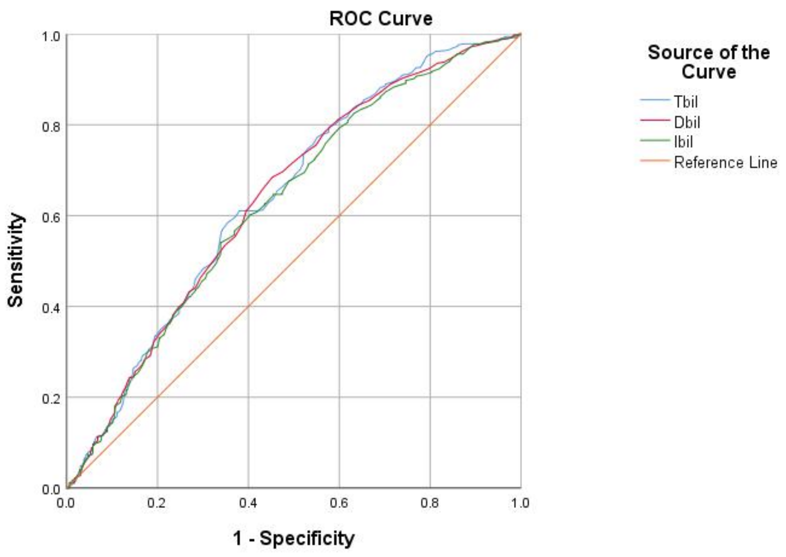

A

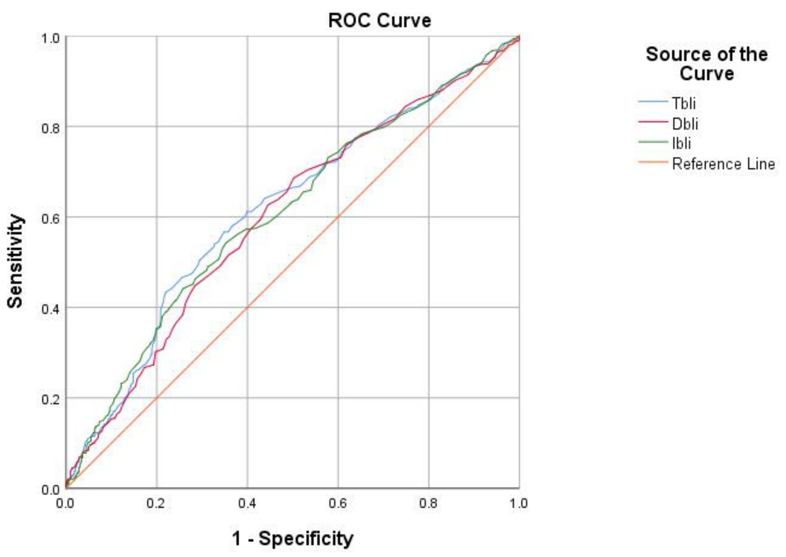

B

Figure S1 ROC curves for bilirubin levels and the extent of ICAS.

(A) ROC curves for bilirubin levels and the arterial stenosis degree of ICAS (combine none-mild and moderate groups together and then compared with severe-occlusion group).

(B) ROC curves for bilirubin levels and the arterial stenosis number of ICAS (combine single- and two-vessel stenosis groups together and then compared with multiple-vessel stenosis group).

Note: ROC, receiver operating characteristic; ICAS, intracranial atherosclerotic stenosis. Tbil: total bilirubin; Dbil: direct bilirubin; Ibil: indirect bilirubin; none-mild group: stenosis 0-49%, moderate group: stenosis 50-69%; severe-occlusion group: stenosis 70-100%.

Table S1 Diagnostic performance of bilirubin levels in ICAS

		AUC (95% CI)	P value	Youden index	Cut-off	Sensitivity (%)	Specificity (%)
Stenosis severity of intracranial artery	Tbli	0.640	<0.0001	0.2305	10.3	61.04	62.01
	Dbli	0.638	<0.0001	0.2319	4.2	68.47	54.71
	Ibli	0.627	<0.0001	0.2013	6.0	54.02	66.11
Number of intracranial artery stenosis	Tbli	0.615	<0.0001	0.2183	10.5	56.67	65.15
	Dbli	0.600	<0.0001	0.1839	3.9	68.58	49.81
	Ibli	0.607	<0.0001	0.1898	5.8	54.21	64.77

Abbreviations: ICAS, intracranial atherosclerotic stenosis; Tbil: total bilirubin; Dbil: direct bilirubin; Ibil: indirect bilirubin; AUC: area under the curve, CI: confidence interval.
